# Emergency Department Urosepsis and Abdominal Imaging

**DOI:** 10.7759/cureus.14752

**Published:** 2021-04-29

**Authors:** Mansoor Siddiqui, Dena Abuelroos, Lihua Qu, Raymond E Jackson, David A Berger

**Affiliations:** 1 Emergency Medicine, Henry Ford Health System, Detroit, MI, USA; 2 Radiology, Beaumont Health, Royal Oak, USA; 3 Research, Beaumont Health, Royal Oak, MI, USA; 4 Emergency Medicine, Beaumont Health, Royal Oak, USA

**Keywords:** em, er, emergency medicine, urosepsis, sepsis, imaging, urinalysis, sirs, source control

## Abstract

Introduction

Insufficient attention has been directed towards urosepsis. Notably, no protocols or clinical decision rules currently exist outlining the appropriate use of imaging in uroseptic patients. The primary objective of our study was to retrospectively evaluate uroseptic emergency department (ED) patients who underwent abdominal imaging, to report the proportion of patients with imaging findings necessitating emergent surgical consultation.

Methods

We retrospectively identified 1142 patients ≥ 18 years of age that presented to the ED from January 2009 to December 2012 with ICD9 code indicative of urosepsis. All included patients underwent ED-ordered abdominal computerized tomography (CT) or retroperitoneal ultrasound (US). Imaging and urinalysis (UA) results were categorized. We report proportions with odds ratios and 95% confidence intervals.

Results

Of 1142 patients, we excluded 80 for neg UA, 167 for < 2 SIRS (systemic inflammatory response syndrome), 320 for positive blood cultures, and 37 for incomplete data. This yielded 538 patients which the authors reviewed the results of the CT or US to determine the proportion who required emergent surgical consultation and who underwent surgical or interventional procedure. There were 243 (45%) that had CT or US results that necessitated emergency surgical consultation, of those 180 (33%) underwent surgical or interventional procedure. Similar rates of emergency surgical consultation occurred when sub-divided by positive versus equivocal UA, with 43% and 47%, respectively.

Conclusions

Forty-five percent of our abdominally imaged urosepsis cohort had imaging findings that necessitated emergent surgical consultation, with a similar proportion in the subset with positive versus equivocal UA. The utility of abdominal imaging in this population should be studied prospectively.

## Introduction

While sepsis morbidity and mortality has decreased markedly, it remained responsible for approximately 11 million global deaths in 2017 [1-5]. Within the United States, sepsis is a leading cause of death with 1.7 million cases and nearly 270,000 deaths per annum [6]. Questions persist regarding the optimal treatment of urosepsis in the emergency department (ED). Although urinary sepsis is often managed with aggressive fluid resuscitation and antibiotic therapy alone, some inciting causes of severe urinary tract infections (UTIs) including ureterolithiasis, nephrolithiasis, and other forms of obstructive uropathy may require surgical source control.

The 2016 Surviving Sepsis Campaign (SSC) guidelines reported with grade 1C recommendation that a “specific anatomic diagnosis of infection requiring consideration for emergent source control should be identified or excluded as rapidly as possible” and the source control procedure occur as soon as practical [7]. While the 2012 SSC guidelines do provide an ungraded recommendation for prompt imaging studies to identify the potential source, this mention of imaging was removed in the 2016 update. Furthermore, a large multi-centered prospective observational study on severe sepsis and septic shock reported that urologic site of infection was the only infection site (when compared to respiratory site) which was associated with a statistically significant reduction in hospital mortality [8].

Abdominal imaging can provide this critical information, however, these studies can be cumbersome and computerized tomography (CT) imaging delivers a dose of radiation [9]. No protocols or clinical decision rules currently exist outlining the appropriate use of imaging in uroseptic ED patients. Similarly, while the interpretation of a clearly negative urinalysis (UA) result and an uncontaminated UA result consistent with UTI are apparent, the clinical relevance of equivocal UA results in uroseptic ED patients has not been established.

The objective of our study was to retrospectively evaluate uroseptic ED patients who underwent abdominal imaging, evaluating for the proportion of patients with imaging findings necessitating emergent surgical consultation. We hypothesized that a substantial proportion of uroseptic ED patients would be found to have imaging results consistent with obstructive uropathy necessitating surgical consultation and surgical or interventional procedure. We also sought to evaluate if positive and equivocal urinalysis results yielded any difference in the rate of emergent surgical consultations. We hypothesized that a UA positive for signs of infection would have a greater likelihood of necessitating emergent surgical consultation than an equivocal UA result.

## Materials and methods

**Figure 1:**
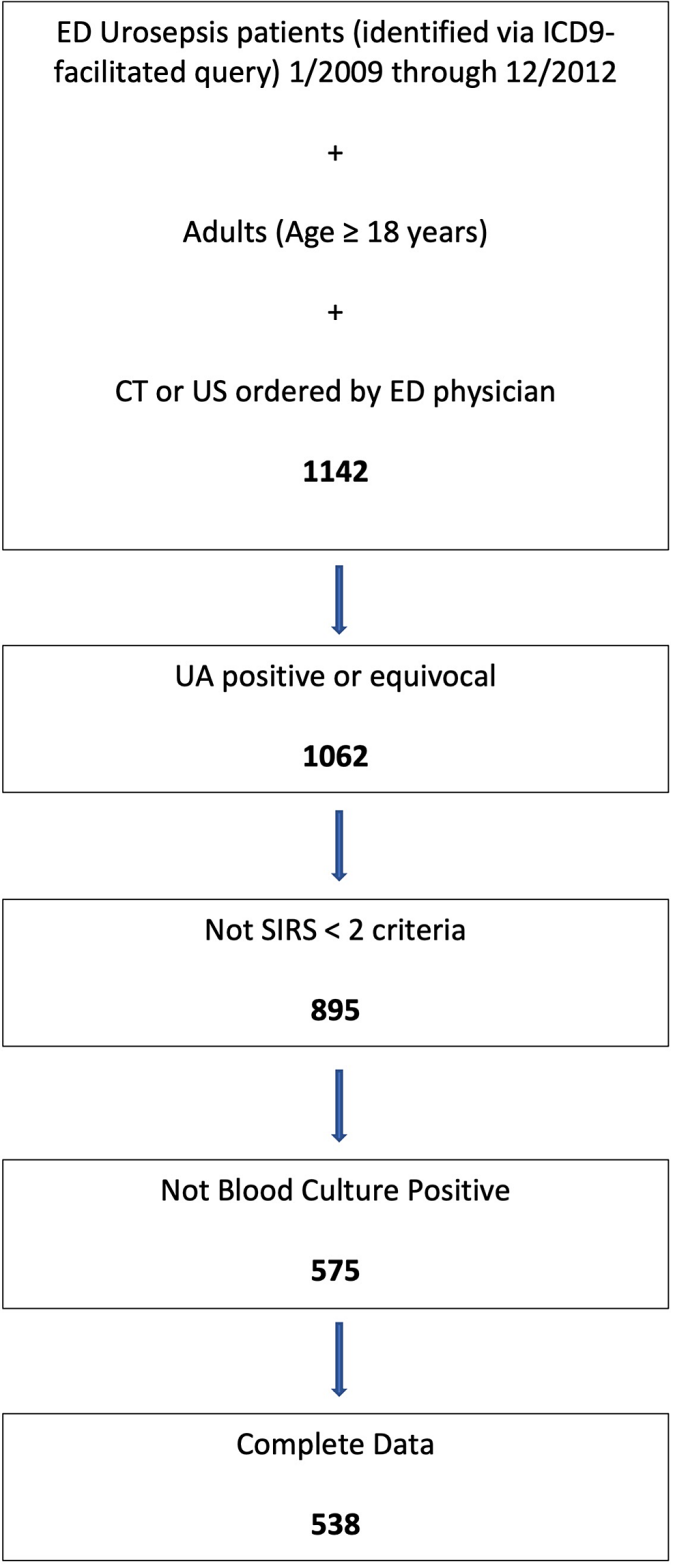
Flow Diagram of ED Urosepsis and Abdominal Imaging Study Participants ED: Emergency Department; ICD: International Classification of Diseases; CT: computerized tomography; US: ultrasound; UA: urinalysis; SIRS: systemic inflammatory response syndrome

We identified adult patients ≥ 18 years of age presenting to the ED between January 2009 and December 2012 with an ICD-9 code for sepsis (995.91) or severe sepsis (995.92) plus urinary tract infection (599.0) or hydronephrosis (591). Patients were included only if they had undergone an abdominal CT scan or retroperitoneal ultrasound (US) of the kidneys ordered by an ED provider (N=1142). CT scans were included with, with/without, and without contrast enhancement. An ad hoc decision was rendered to only include ED urosepsis patients that were imaged. While urosepsis patients that were non-ED imaged, inpatient imaged, or never imaged may be vital to future work, they were not incorporated into this first stage of hypothesis testing. Exclusion criteria are highlighted in Figure 1 and at beginning of the Results section. We notably did exclude patients who had positive blood cultures, since urosepsis can occur secondary to bacteremia and the authors aimed to eliminate this potential confounding factor. These blood culture positive exclusions were ascertained by retrospective chart review, though the culture status was not known to the treating ED physicians.

**Figure 2:**
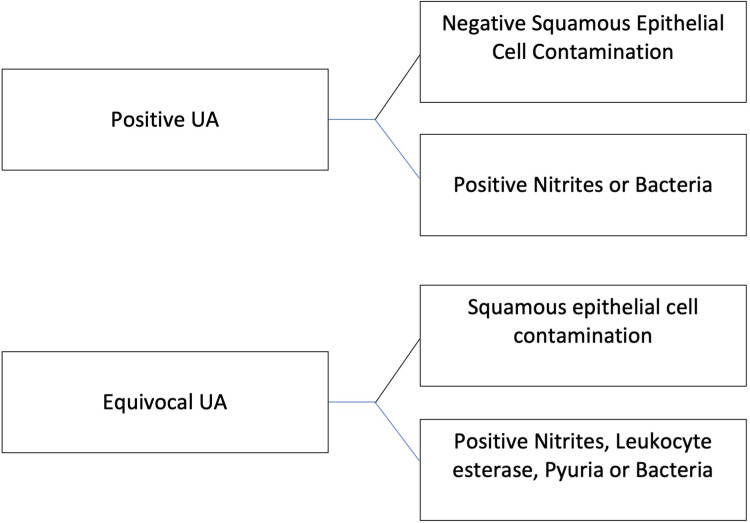
Urinalysis Characteristics to Dichotomize Into Positive Versus Equivocal Results UA: urinalysis

We re-reviewed each patient's urinalysis (UA) to ensure it was equivocal or positive for infection (Figure 2), then analyzed if a patient satisfied two or more systemic inflammatory response syndrome (SIRS) criteria. Two authors (DA, MS) were trained to distinguish among negative, equivocal, and positive UA performed data abstraction, supervised by the senior author (DB). The process of categorizing a UA result is shown graphically in Figure 2 and explained here. A positive UA was defined as the presence of nitrite and/or bacteria without squamous epithelial cell contamination (SECC). We defined an equivocal UA as the presence of one or more of the following with SECC: positive nitrite, positive leukocyte esterase, pyuria, and presence of bacteria. We utilized UA, as opposed to urine culture since the urine culture results would be unknown to the ED providers and a sepsis diagnosis requires a suspected (or present) source of infection.

Radiology imaging reports were evaluated by two of the authors (DA, MS). Based on the imaging results, the patient would be assigned a score of one, two, or three based on the acuity of the finding. The senior author (DB) reconciled differences between the two authors as needed. If an imaging finding required emergent surgical consultation and likely surgical or interventional procedure, the patient was assigned a score of 1. If an emergent surgical consultation was appropriate, however, the patient could likely be managed conservatively without surgical or interventional procedure, the patient was assigned a score of 2. Other non-emergent imaging findings and negative studies were assigned a score of 3. We report proportions with Odds Ratios and 95% confidence intervals. 

## Results

Of the 1142 patients identified via ICD9 query, 538 patients met selection criteria (Figure 1). We excluded patients based on the following criteria: 80 for negative UA; 167 for < 2 SIRS criteria; 320 for positive blood culture; and 37 for incomplete data. 538 patients underwent review of the CT or US findings to determine the percentage who required emergent surgical consultation and the percentage who underwent a surgical or interventional procedure. 

Of the 538 patients, 243 (45%) had abdominal imaging results which indicated the need for emergency surgical consultation, of those 180 (33%) indeed underwent a surgical or interventional procedure and seven (1%) were recommended but declined. The number of patients with obstructive uropathy totaled 189 (35%). An additional 10% had non-urologic imaging findings which necessitated emergency surgical consultation that included cholecystitis, perforated viscus, and bowel obstruction.

Of the 538 patients, 245 (46%) had positive UA of which 105 (43%) required emergent surgical consultation. Of the 293 (54%) equivocal UA patients, 138 (47%) required emergent surgical consultation. The odds ratio of a positive UA (when compared to equivocal UA) requiring emergent surgical consultation (Table 1) is 0.84 (95% CI 0.6 to 1.19).

**Table 1:** Odds Ratio of Abdominally Imaged ED Urosepsis Patient With Positive UA Requiring Emergent Surgical Consultation Compared to Equivocal UA ED: Emergency Department; UA: urinalysis; CI: confidence intervals

	Positive UA	Equivocal UA
Emergent Surgical Consultation	105	138
No Consultation Required	140	155
Total Patients	245	293
Positive UA Odds Ratio = 0.84 (CI 0.60-1.19)		

## Discussion

The primary findings of our retrospective descriptive study demonstrated that 45% of our abdominally imaged urosepsis cohort had abdominal findings that required emergent surgical consultation and 33% underwent a surgical or interventional procedure. We also report that ED-obtained initial UA (positive vs equivocal) did not differentiate who underwent emergent surgical consultation. Together, these findings suggest that when a urosepsis clinical scenario suggests the need for abdominal imaging, be highly suspicious for imaging findings that may require a surgical or interventional procedure. It further suggests that in this population to not dismiss an equivocal UA.

Sepsis screening has evolved over the last three decades, starting with SIRS, quick sequential organ failure assessment (qSOFA), and now re-interest in SIRS [10-13]. The deployment of sepsis bundles, assessing compliance, and public reporting have been reported to reduce the length of stay and risk-adjusted mortality [14, 15]. Additional studies have evaluated the impact of dedicated staffing [16], electronic medical record alerts [17, 18], and other machine-learning-driven research [19] on sepsis metrics. 

The time to achieve the sepsis bundle has been subjected to significant debate since the establishment of the 1-hour bundle [20]. The debate revolves around the perception that this rush to treat may yield poor sepsis diagnostic specificity and potential for harm from this overtreatment and one-size-fits-all approach [21]. In the context of our trial, urinary sepsis is not always apparent (or even suspected) within “one hour of ED presentation,” and the time of ED presentation is when the sepsis 1-hour clock starts. Moreover, abdominal imaging via CT or US requires patient stability, available transport, and time to attain radiology interpretation. Furthermore, if these variables are in direct conflict with bundle compliance, providers may be indirectly incentivized to delay their search for source control.

In our study, the emergent surgical consultations were obtained in large part for urology evaluation of septic stones and obstructive uropathy, however, 10% of study patients were found to have imaging findings consistent with emergencies seemingly unrelated to urosepsis (cholecystitis, perforated viscus, and bowel obstruction). In this regard, an unknown percent of our cohort may not have been primarily uroseptic but had another type of illness with a concomitantly positive UA. Our study group believes this subset of our cohort is small and therefore does not suspect it negatively impacted our research. There is neonatal literature that examined whether positive urinalysis is indicative of UTI or if the UA is actually a tool to facilitate risk stratification of serious bacterial infections [22, 23]. The author has not been able to discover studies evaluating this same principle or efforts towards decision rules with adults. An ED provider’s understanding of plainly positive and negative UAs is clear; however, the relevance of an equivocal result is unclear. Our study reveals no statistically significant difference between positive and equivocal UA results in the subset of uroseptic ED patients requiring emergent surgical consultation, highlighting the clinical relevance of an equivocal UA. 

Bloos et al. reported with a cohort of 422 patients who underwent source control, no association between source control timing and 28-day mortality [24]. However, in their entire cohort (N=1011), patients who had source control within six hours were associated with a 16% mortality reduction. While there have been research endeavors of source control interventions and timing, there is no literature known to our authors on the optimal strategy (or imaging required) to diagnose the surgical source. This is a very significant gap in sepsis management. Our study highlights the need to perform a randomized controlled trial (RCT) to further investigate abdominal imaging in urosepsis and develop clinical decision rules so that the imaging is utilized appropriately. 

The authors acknowledge that our study had multiple limitations. With respect to methodology, it was a retrospective, single-site, convenience sample, and patients were identified via an electronic medical record, ICD9-facilitated query. Furthermore, there is not an ICD9 or ICD10 code for specifically titled “urosepsis,” which hampers a straightforward ICD-driven search strategy. There is also selection bias since we cannot ascertain the reason the abdominal imaging study was ordered, or account for patients who were imaged after admitted to the hospital. While not known with certainty, it is possible that abdominal imaging was more likely to be ordered in higher acuity patients and thereby generated a higher proportion of imaging positivity (when compared to a study of all ED uroseptic patients).

Our exclusion criteria were intended to be conservative, very evident by our exclusion of patients that had positive blood cultures. These were eliminated from our cohort to minimize the possibility that bacteremia spread hematogenously to the kidneys, which would conflict with our intention to analyze sepsis cases that appear to have primary urinary causation. It is unknown how this ad hoc decision has impacted our results.

Although UA interpretation is somewhat subjective, we used strict criteria to evaluate UA results as outlined in the Methods section above and feel the use of subjective interpretations would create negligible bias. In contrast, the authors feel that our decision to include both positive and equivocal UA was a strength of our study. 

At the time of this study in 2009-2012, SIRS a key component of sepsis definitions [10] which is why we used this to generate our cohort. By excluding 167 patients with < 2 SIRS criteria, the authors recognize the non-inclusion of ED-cases who may have satisfied qSOFA or later developed ≥ 2 SIRS criteria while in hospital. Our study duration also pre-dated the popularization of Centers for Medicare and Medicaid Services (CMS) sepsis core measures and increasing attention to the Surviving Sepsis Campaign. It also pre-dated the ED usage of procalcitonin (PCT) for sepsis diagnosis and ICU usage for sepsis antibiotic de-escalation. Procalcitonin in febrile UTI has been investigated to predict the presence of bacteremia [25], though this marker was not utilized at our institution during our study period.

## Conclusions

We observed that 45% of our abdominally imaged urosepsis cohort had imaging findings that required emergent surgical consultation and 33% underwent surgical intervention. We further demonstrated that a positive and equivocal urinalysis were associated with similar rates of emergent surgical consultation. Together, these findings suggest that when a urosepsis clinical scenario suggests the need for abdominal imaging, be highly suspicious for imaging findings that may require a surgical or interventional procedure. It further suggests that in this population to not dismiss an equivocal UA. The utility of abdominal imaging in this population should be studied prospectively.

## References

[1] Rivers E, Nguyen B, Havstad S (2001). Early goal-directed therapy in the treatment of severe sepsis and septic shock. N Engl J Med.

[2] Yealy DM, Kellum JA, Huang DT (2014). A randomized trial of protocol-based care for early septic shock. N Engl J Med.

[3] Peake SL, Delaney A, Bailey M (2014). Goal-directed resuscitation for patients with early septic shock. N Engl J Med.

[4] Mouncey PR, Osborn TM, Power GS (2015). Trial of early, goal-directed resuscitation for septic shock. N Engl J Med.

[5] Rudd KE, Johnson SC, Agesa KM (2020). Global, regional, and national sepsis incidence and mortality, 1990-2017: analysis for the Global Burden of Disease Study. Lancet.

[6] (2021). Healthcare Professional Information - Center for Disease Control and Prevention. https://www.cdc.gov/sepsis/education/hcp-resources.html.

[7] Rhodes A, Evans LE, Alhazzani W (2017). Surviving Sepsis Campaign: International Guidelines for Management of Sepsis and Septic Shock: 2016. Intensive Care Med.

[8] Martínez ML, Ferrer R, Torrents E (2017). Impact of source control in patients with severe sepsis and septic shock. Crit Care Med.

[9] Brenner DJ, Hall EJ (2007). Computed tomography--an increasing source of radiation exposure. N Engl J Med.

[10] Bone RC, Balk RA, Cerra FB (1992). Definitions for sepsis and organ failure and guidelines for the use of innovative therapies in sepsis. The ACCP/SCCM Consensus Conference Committee. American College of Chest Physicians/Society of Critical Care Medicine. Chest.

[11] Seymour CW, Liu VX, Iwashyna TJ (2016). Assessment of clinical criteria for sepsis: for the Third International Consensus Definitions for Sepsis and Septic Shock (Sepsis-3). JAMA.

[12] Gando S, Shiraishi A, Abe T (2020). The SIRS criteria have better performance for predicting infection than qSOFA scores in the emergency department. Sci Rep.

[13] Serafim R, Gomes JA, Salluh J, Póvoa P (2018). A comparison of the quick-SOFA and systemic inflammatory response syndrome criteria for the diagnosis of sepsis and prediction of mortality: a systematic review and meta-analysis. Chest.

[14] Levy MM, Gesten FC, Phillips GS (2018). Mortality changes associated with mandated public reporting for sepsis. The results of the New York state initiative. Am J Respir Crit Care Med.

[15] Afshar M, Arain E, Ye C (2019). Patient outcomes and cost-effectiveness of a sepsis care quality improvement program in a health system. Crit Care Med.

[16] Burla MJ, Shinthia N, Boura JA, Qu L, Berger DA (2020). Resuscitation resident impact in the treatment of sepsis. Cureus.

[17] Seetharaman S, Wilson C, Landrum M (2019). Does use of electronic alerts for systemic inflammatory response syndrome (SIRS) to identify patients with sepsis improve mortality?. Am J Med.

[18] Narayanan N, Gross AK, Pintens M, Fee C, MacDougall C (2016). Effect of an electronic medical record alert for severe sepsis among ED patients. Am J Emerg Med.

[19] Delahanty RJ, Alvarez J, Flynn LM, Sherwin RL, Jones SS (2019). Development and evaluation of a machine learning model for the early identification of patients at risk for sepsis. Ann Emerg Med.

[20] Levy MM, Evans LE, Rhodes A (2018). The surviving sepsis campaign bundle: 2018 update. Crit Care Med.

[21] Spiegel R, Farkas JD, Rola P, Kenny JE, Olusanya S, Marik PE, Weingart SD (2019). The 2018 surviving sepsis campaign's treatment bundle: when guidelines outpace the evidence supporting their use. Ann Emerg Med.

[22] Wang ME, Biondi EA, McCulloh RJ, Garber MD, Natt BC, Lucas BP, Schroeder AR (2019). Testing for meningitis in febrile well-appearing young infants with a positive urinalysis. Pediatrics.

[23] Kuppermann N, Dayan PS, Levine DA (2019). A clinical prediction rule to identify febrile infants 60 days and younger at low risk for serious bacterial infections. JAMA Pediatr.

[24] Bloos F, Thomas-Rüddel D, Rüddel H (2014). Impact of compliance with infection management guidelines on outcome in patients with severe sepsis: a prospective observational multi-center study. Crit Care.

[25] van Nieuwkoop C, Bonten TN, van't Wout JW (2010). Procalcitonin reflects bacteremia and bacterial load in urosepsis syndrome: a prospective observational study. Crit Care.

